# Clinical Utility of Gene Expression Profiling Data for Clinical Decision-Making Regarding Adjuvant Therapy in Early Stage, Node-Negative Breast Cancer: A Case Report

**DOI:** 10.3390/jpm2030071

**Published:** 2012-09-10

**Authors:** Steven R. Schuster, Barbara A. Pockaj, Mary R. Bothe, Paru S. David, Donald W. Northfelt

**Affiliations:** 1 Division of Hematology and Oncology, Mayo Clinic Arizona, 13400 E Shea Blvd., Scottsdale, AZ 85259, USA; E-Mails: bothe.mary@mayo.edu (M.R.B.); Northfelt.donald@mayo.edu (D.W.N.); 2 Department of Surgery, Mayo Clinic Arizona, 13400 E Shea Blvd., Scottsdale, AZ 85259, USA; E-Mail: pockaj.barbara@mayo.edu; 3 Department of Womens Health, Mayo Clinic Arizona, 13400 E Shea Blvd., Scottsdale, AZ 85259, USA; E-Mail: david.paru@mayo.edu

**Keywords:** breast cancer, genomic profiling, medical decision-making, Oncotype DX, chemotherapy, personalized medicine

## Abstract

Breast cancer is the most common malignancy among women in the United States with the second highest incidence of cancer-related death following lung cancer. The decision-making process regarding adjuvant therapy is a time intensive dialogue between the patient and her oncologist. There are multiple tools that help individualize the treatment options for a patient. Population-based analysis with Adjuvant! Online and genomic profiling with Oncotype DX are two commonly used tools in patients with early stage, node-negative breast cancer. This case report illustrates a situation in which the population-based prognostic and predictive information differed dramatically from that obtained from genomic profiling and affected the patient’s decision. In light of this case, we discuss the benefits and limitations of these tools.

## 1. Introduction

Breast cancer is the most common malignancy among women in the United States with the second highest incidence of cancer-related death following lung cancer [1]. Adjuvant hormonal therapy and chemotherapy are commonly prescribed to reduce the risk for relapse by eliminating microscopic deposits of malignancy that have not been eliminated by primary therapy (surgery and radiation therapy). The value of adjuvant therapy is unpredictable in an individual patient as the risk for relapse varies between patients.

There are multiple tools that provide prognostic data regarding risk for relapse in an individual patient as well as predict the potential benefit of adjuvant systemic therapy. One commonly used tool is Adjuvant! Online [2]. This program utilizes a patient’s objective data, such as tumor size, node involvement and tumor receptor status, to tailor its output. However, the current version of Adjuvant! Online does not include any means for consideration of genomic information derived from the primary tumor. Gene expression profiling (e.g., Oncotype DX) can provide prognostic and predictive information based on the genomic characteristics of the patient’s tumor [3]. This creates the potential for disagreement between the population-based analysis and genomic-based information that can confound decision-making regarding adjuvant therapy.

The following case report illustrates a situation in which the population-based prognostic and predictive information differed dramatically from that obtained from genomic profiling.

## 2. Case Presentation

A healthy 58 year old post-menopausal woman with no family history of breast or ovarian cancer presented after screening mammography found a new 0.7 cm cluster of microcalcifications in the tail of the left breast. Left breast biopsy revealed a 1.0 cm infiltrating ductal carcinoma with medullary features, Nottingham grade 3. Immunohistochemical staining demonstrated expression of estrogen receptor, no expression of progesterone receptor, and no HER2 over-expression. Sentinel node sampling revealed no metastatic disease.

**Figure 1:**
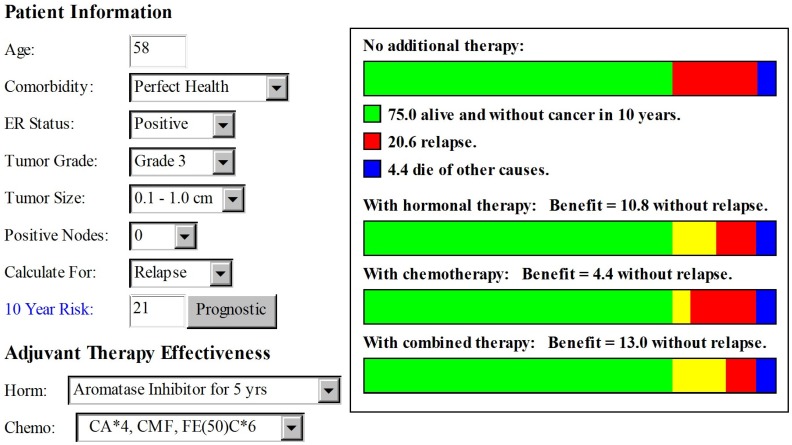
Prognostic data based on population-based analysis with Adjuvant! Online version 8.0 [4].

Prognostic information from Adjuvant! Online was as shown in Figure 1. The patient’s likelihood of remaining free of disease relapse in the next 10 years was reported to be 75% if no adjuvant treatment was prescribed. The addition of adjuvant aromatase inhibitor therapy would increase the likelihood of remaining disease free to 85.8% at 10 years, an absolute increase of 10.8%. Adding adjuvant chemotherapy would further increase the likelihood of remaining free of relapse by an additional 2.2%. Based on these predictions, the patient was inclined to receive only hormonal therapy and forego adjuvant chemotherapy with its potential for significant toxicity in return for only a modest gain in reducing relapse risk. 

**Figure 2:**
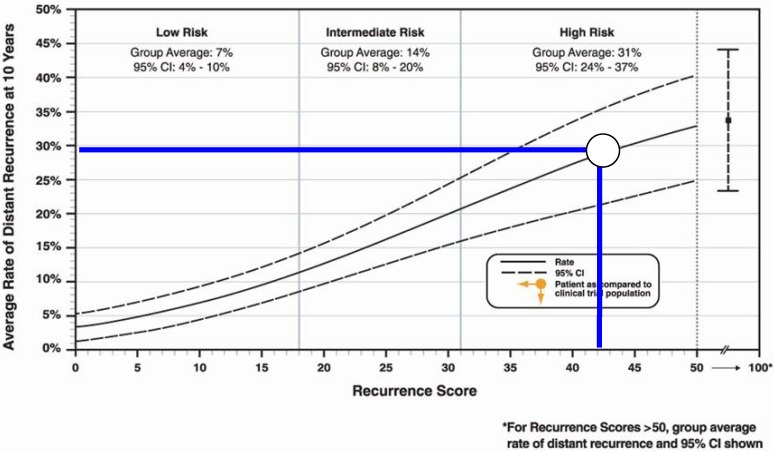
Risk of distant recurrence at 10 years based on genomic analysis with Oncotype DX. Patient recurrence score = 42.

However, the patient’s tumor specimen was also sent for genomic profiling using Oncotype DX (Figure 2). This analysis revealed a recurrence score of 42 corresponding to a 69% likelihood of remaining free of relapse at 10 years if the patient was treated only with adjuvant hormonal therapy (tamoxifen), an absolute difference of 16.8% compared to the population based prediction.

**Figure 3:**
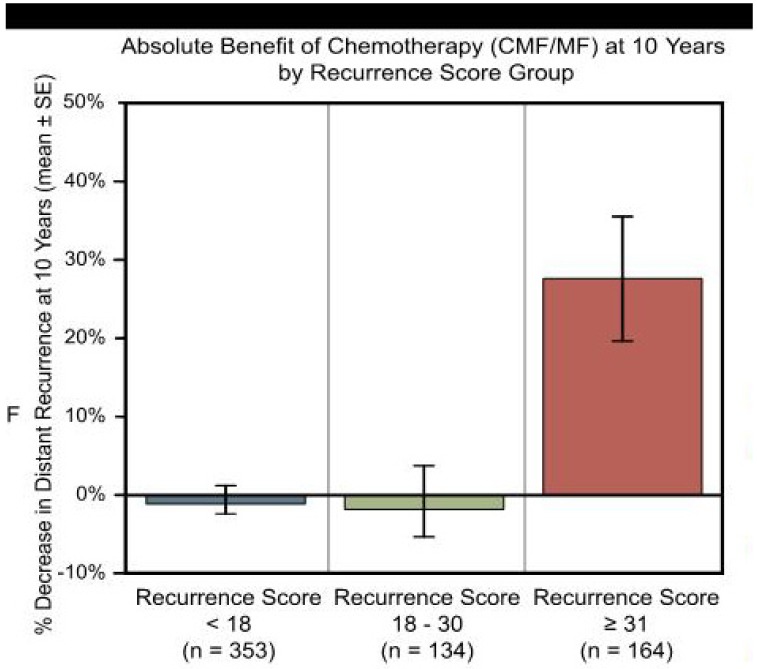
Demonstration of benefit of chemotherapy in patients in high risk group based on genomic analysis.

After extensive discussion, the patient expressed concern over the less optimistic prediction of the genomic profiling assessment. She therefore elected to pursue adjuvant chemotherapy with cyclophosphamide, doxorubicin, and paclitaxel to obtain the predicted added benefit of chemotherapy (Figure 3). The patient tolerated the treatment well with some peripheral neuropathy, and she currently has no evidence of disease 36 months after her adjuvant chemotherapy.

## 3. Discussion

The classic population-based variables considered by Adjuvant Online! include age, presence of comorbidities, estrogen receptor expression status, histologic grade of the tumor, tumor size, number of lymph nodes containing metastatic deposits, and type of adjuvant therapy to be administered [2]. Population-based data from randomized, prospective clinical trials, as compiled and summarized by the Early Breast Cancer Trialists’ Collaborative Group and other sources, are then applied to the patient-specific information to derive prognostic and predictive information that can help the individual patient and her oncologist make informed decisions regarding adjuvant therapy.

Gene expression analysis by Oncotype DX evaluates an individual tumor for 21 specific genes to produce the recurrence score (RS) [5]. This score has been shown to be prognostic for breast cancer recurrence and predictive of adjuvant chemotherapy benefit [3,5,6]. Furthermore, an additional study demonstrated the association between the RS and risk of breast cancer death [7]. Use of this particular genomic analysis tool led to changes in decision-making in 31%–44% of patients based on two studies [8,9].

Both population-based analysis and genomic analysis have limitations. Population-based analysis may lead to over-treatment of low risk patients and under-treatment of high-risk patients. While the RS is divided into three risk groups (low, intermediate, and high), only the high risk group demonstrates significant benefit in risk of relapse from adjuvant chemotherapy (see Figure 3) [6]. This creates a potential for confusion in decision-making, as patients may place too much weight on the concept of ‘intermediate risk’ where the optimum treatment is unknown. The patient may choose an overly aggressive treatment regimen with its associated toxicity due to this uncertainty. In order to determine the best treatment strategies in this ‘intermediate risk’ group, a randomized trial, the Trial Assigning Individualized Options for Treatment (TAILORx), is underway comparing adjuvant chemotherapy and hormonal therapy to hormonal therapy alone in this patient population [10]. Another confounding factor is that studies validated Oncotype DX retrospectively, whereas Adjuvant! Online is based on randomized, prospective trials.

In this case, both the Adjuvant! Online population-based analysis and the Oncotype DX genomic analysis were employed to determine prognosis and predict the benefit of adjuvant systemic therapy to reduce relapse risk. Whereas the initial population-based analysis led the patient to decide against adjuvant chemotherapy, she reversed that decision and elected to pursue adjuvant chemotherapy in addition to adjuvant hormonal therapy once the genomic analysis demonstrated that her risk for relapse was higher, and predicted benefit of chemotherapy correspondingly greater, than had been suggested by the population-based analysis.

The decision-making process is a time intensive dialogue between the patient and her oncologist. Adjuvant! Online and Oncotype DX are tools intended to supplement this dialogue that will develop and improve with time [11]. Numerous variables, such as the patient’s value system, play critical roles.
